# Negative-pressure wound therapy in combination with bronchial occlusion to treat bronchopleural fistula: a case report

**DOI:** 10.1186/s40792-021-01144-4

**Published:** 2021-03-02

**Authors:** Masashi Iwasaki, Masanori Shimomura, Tsunehiro Ii

**Affiliations:** 1 Department of General Thoracic Surgery, Ayabe City Hospital, 20-1 Otsuka, Aono-cho, Ayabe, Kyoto 623-0011 Japan; 2 Division of Thoracic Surgery, Department of Surgery, Graduate School of Medical Science, Kyoto Prefectural University of Medicine, 465 Kajii-cho, Kawaramachi-Hirokoji, Kamigyo, Kyoto 602-8566 Japan

**Keywords:** Negative-pressure wound therapy, Bronchopleural fistula, Open window thoracostomy, Bronchial occlusion

## Abstract

**Background:**

Bronchopleural fistula, which usually accompanies bronchial fistula and empyema, is a severe complication of lung cancer surgery. Negative-pressure wound therapy can enhance drainage and reduce the empyema cavity, potentially leading to early recovery. This therapy is not currently indicated for bronchopleural fistulas because of the risk of insufficient respiration due to air loss from the fistula.

**Case presentation:**

A 73-year-old man, who was malnourished because of peritoneal dialysis, was referred to our hospital for the treatment of lung cancer. Right lower lobectomy with mediastinal lymph node dissection was performed via posterolateral thoracotomy, and the bronchial stump was covered with the intercostal muscle flap. His postoperative course was uneventful and he was discharged. However, he was readmitted to our hospital because of respiratory failure and diagnosed as having bronchopleural fistula on the basis of the bronchoscopic finding of a 10-mm hole at the membranous portion of the inlet of the remnant lower lobe bronchus. Thus, thoracotomy debridement and open window thoracostomy were immediately performed. After achieving infection control, bronchial occlusion was performed using fibrin glue and a polyglycolic acid sheet was inserted through a fenestrated wound. Bronchial fistula closure was observed on bronchoscopy; therefore, a negative-pressure wound therapy system was applied to close the fenestrated wound. The collapsed lung was re-expanded and the granulation tissue around the wound increased; therefore, thoracic cavity size decreased and thoracoplasty using the latissimus dorsi was performed.

**Conclusions:**

This bronchopleural fistula was treated successfully after a right lower lobectomy using an extra-pleural bronchial occlusion and negative-pressure wound therapy.

## Background

According to the National Clinical Database, bronchopleural fistula (BPF) is a severe complication of lung cancer surgery, occurring at a frequency of 0.3%–0.94%, and is associated with a 22%–35.7% mortality rate [1, 2]. BPF treatment involves infection control by fenestration and drainage of the pleural cavity, followed by fistula closure. Negative-pressure wound therapy (NPWT) accelerates wound healing, improves residual lung tissue re-expansion, and narrows the empyema cavity [3]. However, NPWT has not been examined comprehensively as a treatment for empyema with fistula [4].

Here, we present a case of successful BPF treatment after a right lower lobectomy using a combined treatment with extra-pleural bronchial occlusion and NPWT.

## Case presentation

**Fig. 1:**
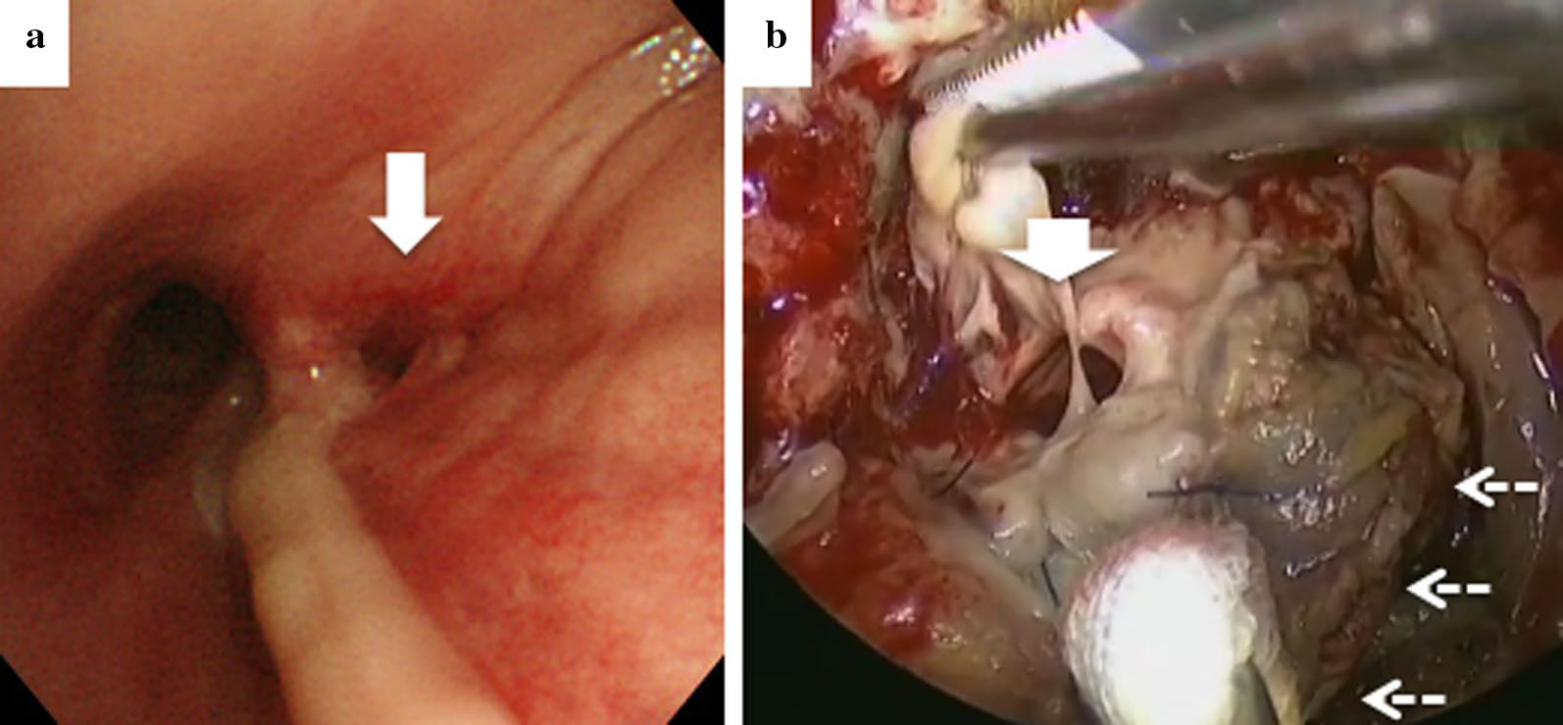
**a** Bronchoscopic and **b** intraoperative findings, showing a 10-mm hole (white arrow) at the membranous portion of the edge of the lower bronchus (dashed arrows)

A 73-year-old male with a 15-month history of peritoneal dialysis for chronic renal failure came to our hospital because of a 3.6-cm squamous cell carcinoma of the right lower lobe. He was malnourished, with a serum albumin level of 2.7 g/dL and a body mass index of 23.0 kg/m^2^. The patient underwent a right lower lobectomy with mediastinal lymph node dissection via a posterolateral thoracotomy. During lymph node dissection, we confirmed the course of the bronchial artery, and dissected the bronchial artery for systematic inferior mediastinal dissection. The bronchial stump was covered with the sixth intercostal muscle flap. Pathological staging was pT1cN0M0 stage IA3. He was uneventfully discharged on postoperative day (POD) 6 but readmitted with respiratory failure on POD 21. Bronchoscopy revealed a 10-mm hole at the membranous portion of the inlet of the remnant lower lobe bronchus (Fig. 1a).

Thoracotomy debridement was immediately performed, revealing foul-smelling pus confined around the fistula and a 10-mm hole at the membranous portion near the stump of the lower bronchus (Fig. 1b). An open window thoracostomy (OWT) was performed considering the patient’s poor general condition. Direct closure of the fistula was not indicated; therefore, we used the second intercostal muscle flap as a patch. Regarding this flap, the seventh rib was dissected and the seventh intercostal muscle flap was collected. We attached the muscle flap to the bronchial fistula at four locations surrounding the fistula by using 4-0 polydioxanone monofilament sutures in a horizontal mattress technique (Ethicon, Somerville, USA), and the entire fistula was covered with the muscle flap.

After OWT, we started to exchange the intrathoracic gauze and antibiotic therapy with meropenem for *Pseudomonas aeruginosa*. After *Candida albicans* and methicillin-resistant *Staphylococcus epidermidis* were detected in the pus culture, micafungin and vancomycin were chosen owing to their drug sensitivity levels. As the patient was receiving peritoneal dialysis, micafungin therapy was administered at a dose of 150 mg/day, and vancomycin therapy was given at a dose and interval of 0.5 g/2 days. As a result, infection control was achieved.

Owing to the prolonged intensive care unit stay, the patient had insomnia, for which we administered a hypnotic drug. However, he developed CO_2_ narcosis due to the hypnotic drug and was temporarily ventilated. Mechanical ventilation resulted in CO_2_ discharge from the body and revealed a large amount of air leakage from the BPF.

**Fig. 2:**
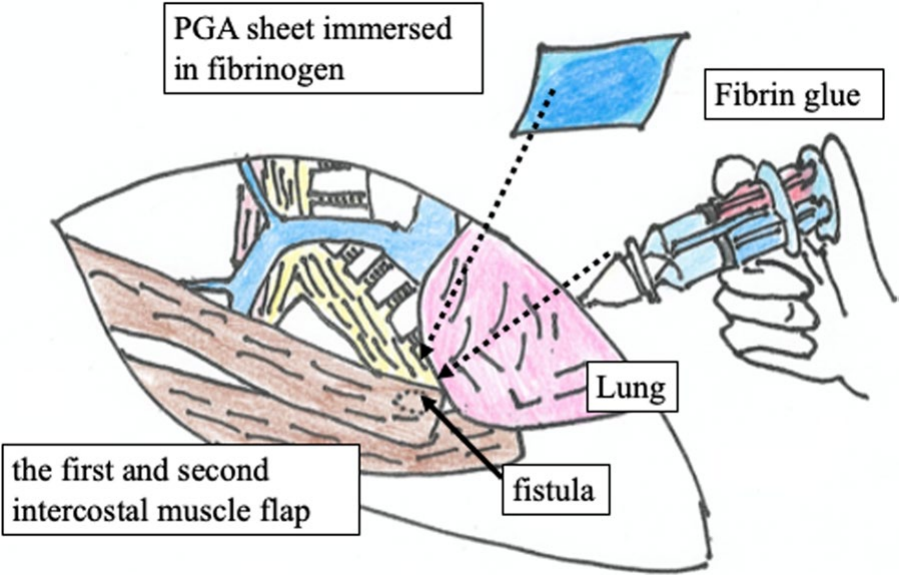
The extra-pleural bronchial occlusion technique. The gap between the fistula and the muscle flap was filled with folded PGA sheets immersed in fibrinogen, and fibrin glue was then spread above the sheets through a fenestrated wound

**Fig. 3:**
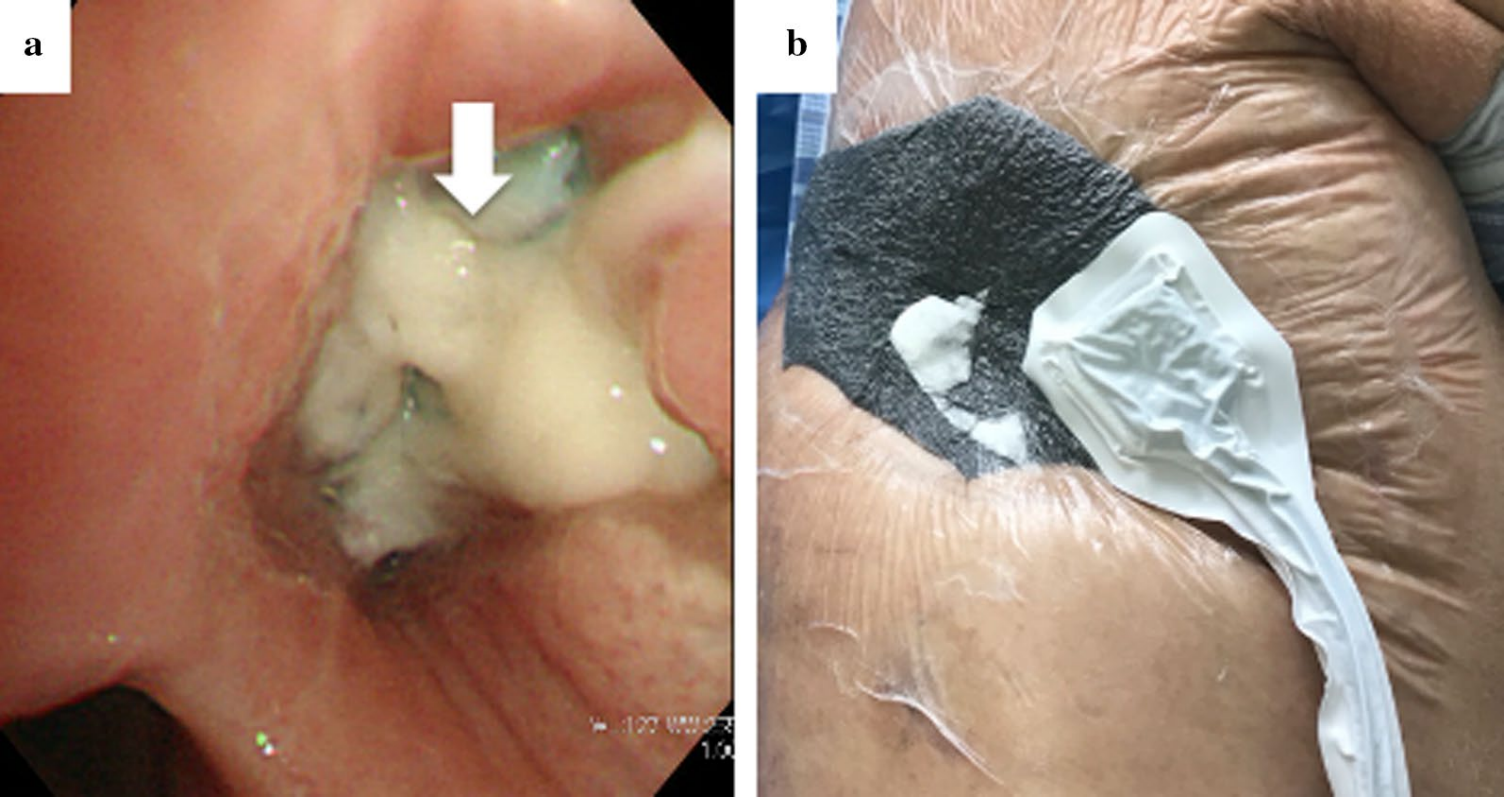
**a** Bronchoscopy findings. After performing successful bronchial occlusion using a fibrin glue sealant and polyglycolic acid sheet, the bronchial fistula is closed. The white arrows indicate that the fistula was covered with granulation tissue. **b** The negative-pressure wound therapy (NPWT) system. The technique entails placing a wound sponge on the fenestrated wound, sealing the site with an adhesive drape, and applying negative pressure at 80 mmHg

**Fig. 4:**
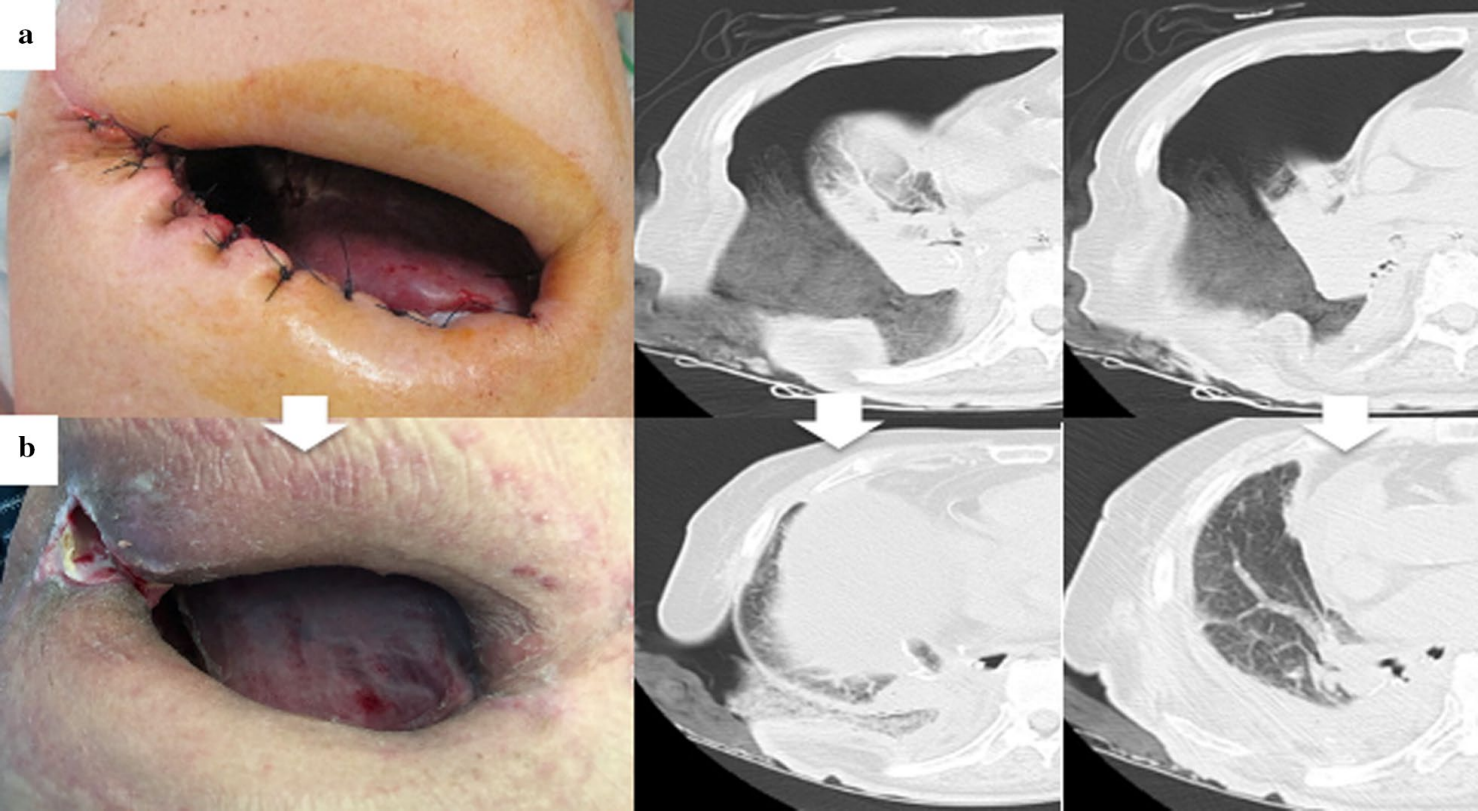
Within 2 months of the open window thoracostomy (OWT) (from **a** to **b**), the pleural cavity gradually decreased in size; the granulation around the wound increased, and the residual lung expanded

Four weeks after the OWT, the culture of the intrathoracic effusion was negative and the thoracic cavity was cleaned. Thus, we performed the following bronchial occlusion procedure: since the patient was treated under mechanical ventilation for CO_2_ narcosis, we used sedating and analgesic drugs, and performed bronchial occlusion procedure in 20 min under direct vision at bedside, in a sterile environment. We attempted to close the BPF using a fibrin glue sealant and polyglycolic acid (PGA) sheet from the extra-pleural side (i.e., the extra-pleural bronchial occlusion technique). The PGA sheet was cut into 5-cm^2^ sheets by immersion in a fibrinogen solution, and the sheets were folded to fill the gap between the fistula and the muscle flap through a fenestrated wound; fibrin glue was then spread above the sheets (Fig. 2). The air leak that was detected earlier in the ventilator circuit had stopped 24 h after the bronchial occlusion. Eighteen days after the bronchial occlusion, a bronchoscopy confirmed complete closure of the fistula (Fig. 3a). Thus, the NPWT system was applied to close the fenestrated wound (Fig. 3b). To avoid direct suction pressure on the lung and other organs, we used a combination of RENASYS-F foam and NPWT system (RENASYS-F® and RENASYS-GO®, Smith & Nephew medical limited, Hull, United Kingdom). The cavity was filled with the RENASYS-F foam; two stacked foams were placed on the lung, covering the mediastinal organs. The wound was sealed with a transparent adhesive drape and connected to the NPWT system. Negative pressure was initiated with a low-pressure level to check for organ effects and gradually increased to 80 mmHg. Four weeks of NPWT treatment resulted in successful re-expansion of the collapsed lung, increased granulation tissue around the wound, and narrowing of the thoracic cavity space (Fig. 4a, b). Finally, we performed thoracoplasty 123 days after the OWT. For thoracic molding, we detached the upper part of the latissimus dorsi muscle from the chest wall, passivated the dorsal axis, and filled the cephalic thoracic cavity. Next, we dissected the eighth and ninth ribs, and removed and detached the thickened wall pleura, filling the remnant cavity above the diaphragm. In addition, we detached the lower part of the latissimus dorsi muscle from the dorsal side and filled the dorsal caudal side of the remnant cavity. Thus, we closed the thoracic cavity by filling the space of the remnant cavity with thickened pleura and vastus lateralis muscle. No recurrence of lung cancer or BPF occurred; the patient died 8 months after the initial surgery due to gastrointestinal bleeding.

## Discussion

The BPF can be closed with several surgical and endoscopic procedures, including suturing of the fistula and intrathoracic transposition of the omentum [5] or muscle flaps [6], and application of a septal occluder device and fibrin glue by bronchoscopy [7]. Such procedures are often unsuccessful and may take months or years to complete. To the best of our knowledge, this is the first reported case of a successful combined-modality treatment involving the extra-pleural bronchial occlusion technique and NPWT for a BPF.

NPWT is a therapeutic option for empyema. Palmen et al. studied NPWT treatment for empyema [3]. Overall, 19 patients underwent OWT for empyema; 8 were treated with conventional OWT, and 11 were treated with NPWT. In the OWT group, the total duration of OWT was 933 days. In the NPWT group, the entire duration of the OWT and NPWT therapies were 39 and 31 days, respectively. However, NPWT is not indicated for the treatment of fistulas, including BPF, because of the risk of organ damage due to the excessive negative pressure through the fistula [4].

Although NPWT was not indicated for the treatment of fistula, cases of successful NPWT for BPF have been reported. Sziklavari et al. reported two cases of detectable BPF treated with NPWT [8]. According to their report, a 1-mm BPF was cured, an 8-mm fistula did not close, and no serious events were observed when NPWT was used with residual BPF or lung tissue at a negative pressure of 75–125 mmHg. In our case, the fistula was 10 mm in diameter and NPWT alone was not expected to close the fistula, so we performed an extra-pleural bronchial occlusion before the NPWT.

After closing the fistula using the extra-pleural bronchial occlusion technique with a fibrin glue sealant and PGA sheet, the chest cavity was progressively reduced using NPWT. We chose the extra-pleural approach to close the fistula because it was large and easily accessible through the fenestrated wound. The addition of fibrin glue sealant minimized air leaks through the PGA sheet.

There is a report of a BPF treatment that combined NPWT and bronchial closure from the intrathoracic side, namely the *intrathoracic bronchial occlusion technique* [7]. Passera et al. used a new cardiovascular device, an Amplatzer (St. Jude Medical, Inc; St. Paul, Minn) double-disk atrial septal defect occlusion tool [7]. Using endobronchial valves followed by fibrin glue, the BPF was successfully closed. When the extra-pleural bronchial occlusion technique failed, the intrathoracic bronchial occlusion technique was considered as an alternative. However, in our case, the bronchial fistula was 10 mm in size, and extra-pleural bronchial occlusion, which could be deformed freely from the outside to close the gap between the fistula and the muscle valve, seemed effective. As a large intrathoracic space remained, we thought that a combination of large-mesh filling and thoracoplasty would be useful, but the patient was receiving peritoneal dialysis, and no biological tissue in the abdominal cavity was available. Filling using other living tissues, such as the vastus lateralis or rectus abdominis muscle, was considered as the next step.

We used a PGA sheet and fibrin glue sealant to close the BPF with an extra-pleural approach. Compared with the former procedures, our extra-pleural bronchial occlusion technique is easier to perform because it does not require a rigid bronchoscope or anesthesia. In cases of light anesthesia, the cough reflex can displace the filling. The PGA sheet can be woven into any shape—depending on the fistula’s configuration—and can be used for fistulas larger than the bronchoscope diameter.

## Conclusions

We successfully combined the extra-pleural bronchial occlusion technique and NPWT in the treatment of BPF. Although NPWT has not been conventionally used to treat empyema with BPF, NPWT may be a good option for patients with empyema and BPF for whom bronchial occlusion has been achieved.

## Data Availability

Not applicable.

## Abbreviations

BPF: Bronchopleural fistula
NPWT: Negative-pressure wound therapy
OWT: Open window thoracostomy
PGA: Polyglycolic acid
POD: Postoperative day
